# The Allergic Rhinitis Clinical Investigator Collaborative – nasal allergen induced eosinophilia

**DOI:** 10.1186/1710-1492-10-S1-A58

**Published:** 2014-03-03

**Authors:** Jenny Thiele, Lisa Steacy, Marie-Eve Boulay, Ann Efthimiadis, Susan Waserman, Paul Keith, Harissios Vliagoftis, Louis-Philippe Boulet, Helen Neighbour, Anne K Ellis

**Affiliations:** 1 Departments of Medicine and Biomedical & Molecular Science, Queen’s University, Kingston, ON, K7L 3N6, Canada; 2 Allergy Research Unit, Kingston General Hospital, Kingston, ON, K7L 2V7, Canada; 3 Institut Universitaire de Cardiologie et de Pneumologie de Quebec, Quebec City, QC, G1V 4G5, Canada; 4 Firestone Institute for Respiratory Health, McMaster University, Hamilton, ON, L8N 4A6, Canada; 5 Department of Medicine, McMaster University, Hamilton, ON, L8N 3Z5, Canada; 6 Pulmonary Research Group, University of Alberta, Edmonton, AB, Canada

## Background

The Allergic Rhinitis Clinical Investigator Collaborative (AR-CIC) is a Canadian multi-center initiative with the primary goal of performing standardized nasal allergen challenge (NAC) to study the anti-allergic effects of novel therapeutic agents for allergic rhinitis (AR). The model further allows identification of potential mechanisms of allergic disease and biomarkers. In this study we examined differential counts, more specifically eosinophil numbers, in nasal lavage samples before, 1 hour (1H) and 6 hours (6H) after direct nasal allergen challenge.

## Methods

Thirty-three atopic and five non-atopic participants were enrolled at four study centers. All atopic participants had AR symptoms following exposure to environmental allergens and a supportive skin test response. Using the Pfeiffer Bidose Nasal Delivery Device 100μl allergen solution was delivered to each nostril. Atopic pilot study participants were challenged with a threshold dose of allergen determined via titration 1 week prior to NAC, non-atopic participants were challenged with a 1:2 allergen dose. The allergens used included either ragweed, grass, D. farina, D. pteronyssinus and cat hair. Nasal lavage samples were collected at baseline, 1H and 6H post NAC. Total cell counts (TCC) were determined on unstained samples prior to cytospin. Cytospin slides were prepared and differentially stained (i.e. DiffQuick).

## Results

Atopic individuals exhibited eosinophilia at 1H and 6H post NAC when compared to baseline samples. Non-atopic participants did not display a significant increase in eosinophils at any time point. Furthermore, TCCs were increased at 1H post NAC in atopic participants. This trend was not observed in non-atopic samples.

## Conclusions

Differences were noted in eosinophil numbers (elevated) between baseline, 1H and 6H post direct NAC only in participants with AR. Nasal lavage collection for differential count analysis is a robust assay that can be integrated into clinical trials conducted using the AR-CIC.

